# Clinical Management of Worn Ball Abutments in Mandibular Mini-Implant Overdentures: A Case Report in a Skeletal Class II Patient

**DOI:** 10.3390/dj13120606

**Published:** 2025-12-16

**Authors:** Cătălina Murariu-Măgureanu, Elena Preoteasa, Cristian Teodorescu, Cristina Teodora Preoteasa

**Affiliations:** 1 Department of Prosthodontics, Faculty of Dentistry, “Carol Davila” University of Medicine and Pharmacy, 020021 Bucharest, Romania; 2 Department of Scientific Research Methods-Ergonomics, Faculty of Dentistry, “Carol Davila” University of Medicine and Pharmacy, 020021 Bucharest, Romania

**Keywords:** mini-implant overdenture, silicone matrices, reconstructive sphere, ball abutments wear, overdenture retention improvement

## Abstract

**Background/Objectives**: Complete denture rehabilitation in edentulous patients presents functional and biomechanical challenges. Mini-implant-supported overdentures improve retention, stability, function, and comfort, particularly in complex class II or class III mandibulo-maxillary relationships. However, mechanical complications such as ball abutment wear may compromise long-term success. This case report aims to describe the clinical context, methods employed to manage ball abutment wear, and related complications in a patient with a mandibular mini-implant overdenture. **Methods**: This retrospective case report presents two approaches to managing abutment wear and enhancing overdenture retention: silicone matrices (Retention.Sil, Bredent Medical GmbH & Co.KG, Senden, Germany) and abutment reconstruction using prefabricated cemented spheres (Concave Reconstructive Sphere, Rhein83, Bologna, Italy). **Results:** A significant mechanical complication associated with mini-implant overdentures is the wear of ball abutments, which may develop over time as a result of continuous interaction between the O-ring system and the abutment surfaces. Both techniques effectively preserved mini-implants while enhancing denture retention, function, and comfort. **Conclusions**: Mechanical complications, such as ball abutment wear, may compromise the retention and functional performance of mandibular overdentures. Alternatives like silicone matrices and reconstructive spheres address abutment wear in mandibular overdentures, ensuring long-term retention and sustainable, patient-centered care for the elderly.

## 1. Introduction

Complete edentulism can be managed through various prosthetic options, including both removable and fixed solutions as well as conventional and implant-supported dentures, each presenting specific advantages and limitations. While complete dentures are commonly provided for elderly individuals with full edentulism, they are associated with numerous functional and biomechanical challenges that can adversely affect oral health-related quality of life [1]. A commonly used option is the implant overdenture, which offers increased stability and mastication efficiency as well as physical and mental comfort for patients. This option is often recommended in more complex cases, such as skeletal class II or III edentulous patients In an edentulous patient exhibiting a skeletal class II jaw relationship, the maxilla is located anterior to the mandible [2]. According to Curtis et al., about 15% of people have a skeletal class II relationship. Treatment is challenging due to skeletal differences and accentuated mandibular movement. The main goal of treatment is to establish a stable, functional bite to minimize overdenture retention complications [3].

There are several options available for implant overdentures, including variations in the number and design of dental implants, attachment systems, and the type of preprosthetic surgery required. When considering this treatment for elderly patients, one subtype that offers specific advantages is the use of mini dental implants. Initially considered a temporary method for overdenture stabilization, these were later adopted as a definitive alternative, particularly in cases such as those involving a narrow mandibular ridge. Their features include avoiding preprosthetic bone augmentation surgery, allowing flapless implant placement, and enabling immediate loading for faster healing and functional restoration.

Overall, mini dental implant overdentures represent a minimally invasive and cost-effective treatment option, frequently resulting in high patient satisfaction among older individuals who are completely edentulous [4]. As with any treatment, complications can develop and should be appropriately managed to maintain favorable outcomes for patients who are already affected by edentulism and the use of dentures. Among these, complications associated with the attachment system, whether ball, bar, magnet, or other types, can compromise prosthesis retention and may contribute to overall failure of the prosthetic restoration [5]. In mini dental implants with a one-piece design, such complications may be more pronounced, as replacement might need to be considered. One issue affecting attachment systems is related to wear [6], frequently reported [7], and the severity varies from mild to severe, as demonstrated in this case report, where we explored different options to improve denture retention and function.

The clinical management of worn ball abutments in mandibular mini-implant overdentures remains insufficiently studied, even though progressive wear reduces retention, stability, and prosthesis function. Although ball attachments are widely used, current literature offers limited guidance on selecting appropriate management strategies. This study is therefore justified by the need for clearer evidence-based protocols to support clinical decision-making and enhance long-term overdenture outcomes, addressing a notable gap in prosthodontic research.

This case report aims to describe the clinical background and the management strategies employed to address ball abutment wear and associated complications in a skeletal class II patient rehabilitated with a mandibular mini-implant overdenture.

## 2. Clinical Case Report: Patient with Skeletal Class II Relationship

A 65-year-old female patient presented to the Clinic of Prosthodontics at “Carol Davila” University of Medicine and Pharmacy, seeking improvement in the function of her mandibular complete denture. The patient had become completely edentulous in the mandible six months prior, following the extraction of her remaining mandibular teeth (lower incisors), and was subsequently fitted with a complete denture. Her primary concern was the instability of the denture during mastication.

### 2.1. Clinical and Radiological Examination

**Figure 1:**
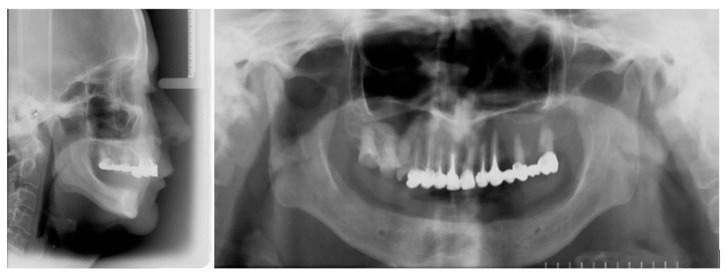
Initial radiological assessment was performed using both lateral cephalometric and panoramic radiographs.

Following clinical and radiological evaluation (Figure 1), the patient was diagnosed with a skeletal class II relationship characterized by a retrognathic mandible, hyperdivergent pattern, and habitual mandibular protrusion during speech. Edentulous spaces in the maxilla were previously restored with fixed dental prostheses, while complete mandibular edentulism was managed with a complete denture.

The previous skeletal class II relationship contributed to the observed large overjet. Additional factors affecting the complexity of the mandibular restoration included uneven residual ridge resorption, thin mucosa covering the ridge, high muscle insertion, bilaterally fibrous retro molar pads, and a reduced support area. A slight increase in vertical dimension of occlusion was noted, accompanied by evidence of patient adaptation such as absence of temporomandibular joint or muscle pain and no deglutition difficulties.

### 2.2. The Implant-Prosthetic Treatment Plan

**Figure 2:**
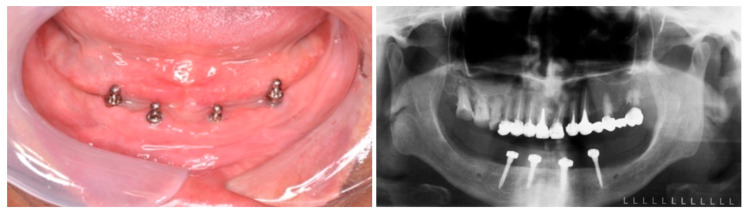
Clinical and radiographic positioning of mini dental implants.

Following the initial assessment and careful consideration of the patient’s perspectives and expectations, the selected treatment plan involved converting the existing complete denture into an implant-retained overdenture by O-ring system. Four Mini^1^ SKY mini dental implants (Bredent, Senden, Germany) were placed in the interforaminal region utilizing a minimally invasive, single-stage flapless technique. The implants featured spherical abutments with an O-ring attachment system, a diameter of 2.8 mm, and lengths of 14 mm for the distal implants and 12 mm for the mesial implants (Figure 2). The insertion torque was recorded as 35 Ncm at level 32 and 25 Ncm at all other sites. Following insertion, no pain or discomfort was reported.

**Figure 3:**
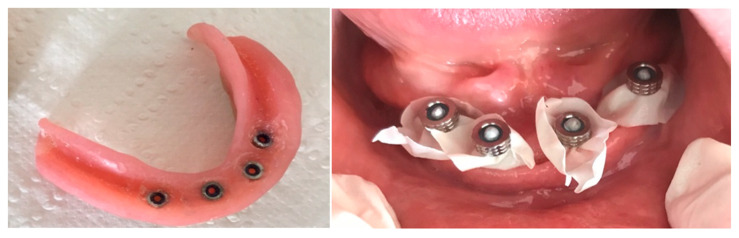
Matrices positioned in the overdenture base (**left**), and the block out of abutments (**right**).

Minor adjustments were made to the complete denture, which was then used as an overdenture. The mandibular overdenture followed a two-step loading protocol, with a soft lining material (Coe Soft, GC America Inc, Alsip, IL, USA) applied for the first three months. Subsequently, the placement of the matrices was carried out. The O-ring matrices (SKY-OR50, Bredent, Senden, Germany) were positioned at the mandibular denture using the direct method with self-polymerizing acrylic resin in the dental clinic. Clinical evaluations were conducted on the internal surface of the denture, including blocking out undercuts on the abutments, assessing adaptation to the mucosa, and performing occlusal analysis (Figure 3).

### 2.3. Fractures of Mandibular Overdentures and the Occurrence of Ball Abutments Wear

**Figure 4:**
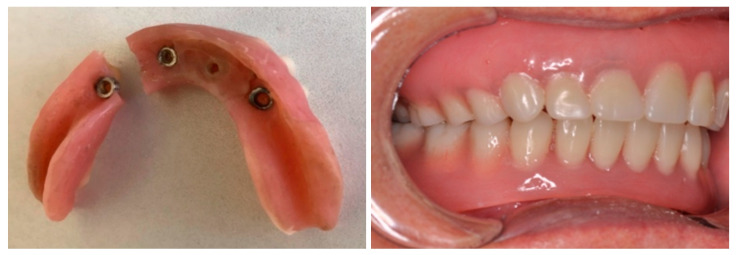
The paramedian fracture line (**left**), and the new overdentures (**right**).

Following the placement of the implants and the application of the overdenture, masticatory function improved, and the implants showed positive progress. After three years, however, the mandibular overdenture experienced a fracture. The fracture line was observed in a paramedian location, adjacent to the matrix of the most distally positioned mini-implant on the right side of the mandible, specifically in an area where the acrylic base exhibited reduced thickness (Figure 4). Simultaneously, loss of the matrix was noted for the mini-implant placed mesially in the left quadrant.

Several factors may have contributed to these complications, including the class II skeletal pattern, a slightly increased vertical dimension of occlusion, a narrow ridge, the presence of antagonistic teeth, and matrix dimensions. The overdenture base was repaired with acrylic resin, and all matrices were replaced chairside using self-polymerizing acrylate. After roughly one year, the mandibular overdenture experienced a second fracture, with the fracture occurring along a line similar to the initial one in the same area of stress concentration, specifically in quadrant 4. Over four years later, the patient experienced multiple dental and prosthetic complications in the maxilla, necessitating the extraction of the affected teeth. A tooth-supported overdenture was subsequently selected as an alternative treatment. At that point, new overdentures were fabricated for both the maxilla and mandible (Figure 4).

During the following five years, the patient received check-ups twice a year for overdenture adjustments, professional cleaning, and assessment of the remaining maxillary teeth and mandibular implants. The progress observed with the new overdentures was positive, and the patient expressed satisfaction regarding improvements in masticatory function, as well as phonetic and aesthetic outcomes. No further incidents of denture instability or insufficient retention were observed; however, the mandibular overdenture fractured again in the same region and was subsequently repaired using metal reinforcement.

**Figure 5:**
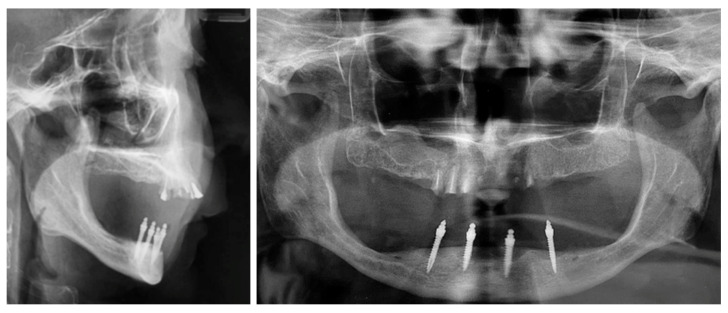
The orthopantomographic and cephalometric radiograph, at 14 years follow-up.

Over a period of approximately ten years, the mandibular overdenture base experienced three fractures at the same location in the fourth quadrant, adjacent to the most distal implant. Additionally, bone resorption was found to be more pronounced at the distal implants compared to the mesial ones (Figure 5). However, clinical examination did not reveal any mobility or other indicators of unfavorable progression.

**Figure 6:**
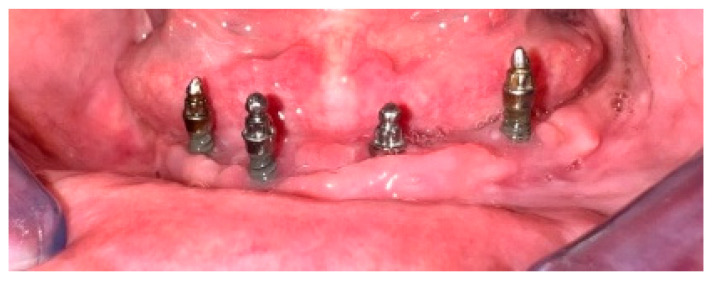
Wear of the spherical abutments.

Another observed mechanical issue was significant wear on the spherical abutments. The distal implants were most impacted, with the abutments altering in shape from spherical to conical (Figure 6). This change may be associated with increased stress levels related to mastication and anterior mandibular movements, as sagittal unbalance.

### 2.4. Prosthetic Alternatives for Managing Complications Associated with Ball Abutment Wear

**Figure 7:**
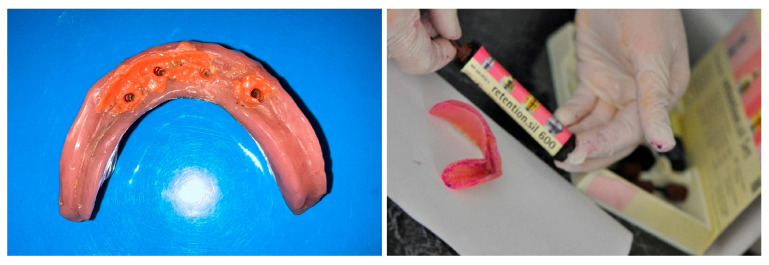
Retention.Sil on overdenture (**left**) and application (**right**).

The complication led to a decrease in overdenture retention because the matrices were no longer secured on the distal abutments, which was attributed to increased wear. The maximum diameter reduction was limited to about 30 μm, and 90% at the equator, due to wear, evaluated with callipers. As a prosthetic approach, a silicone retentive material (Retention.Sil 600, Bredent, Germany) was selected instead of matrices and applied to the internal surface of the overdenture at the site of the mini-implants (Figure 7). The Retention.Sil kit includes one primer and three syringes containing silicones with varying retention levels: 200, 400, and 600. This system proved highly effective in providing retention and stabilization of the overdenture during mastication and phonation, resulting in patient satisfaction. The materials typically remained adherent to the denture for approximately six months before requiring reapplication.

Currently, the maxillary overdenture supported by teeth and the mandibular overdenture on mini-implants fulfilled maintenance requirements, although there were observable signs of artificial tooth wear. The patient noted tooth wear, some chewing difficulty, and denture instability associated with detachment of the silicone matrices.

**Figure 8:**
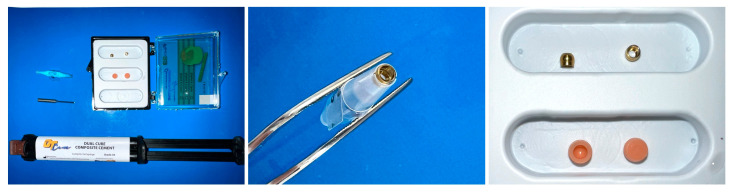
Reconstructive Sphere Kit (**left**), Holder for positioning a sphere on the abutment (**middle**), two spheres and two pink retention caps (**right**).

**Figure 9:**
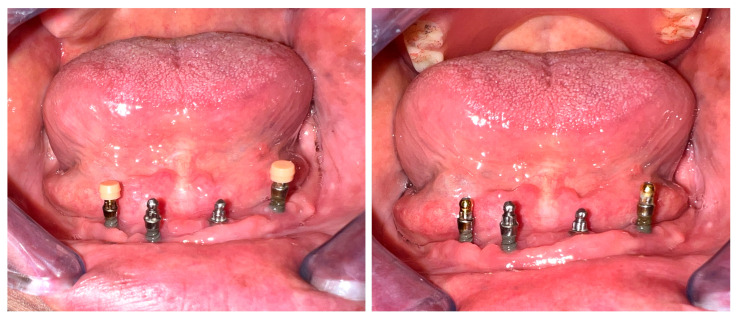
The pink caps over the spheres (**left**), the cemented spheres over the worn abutments (**right**).

In this case, we selected a long-term prosthetic solution using prefabricated reconstructive spheres, cemented with dual cure onto worn abutments (Concave Reconstructive Sphere, Rhein83, Bologna, Italy) (Figure 8). The Reconstructive Sphere kit enables the rebuilding of worn spheres in the mouth to restore long-term retention and stability for dentures using concave titanium spheres. The kits come with spheres in three diameters: 2.5 mm, 2.2 mm, and 1.8 mm. In this case, we used a kit containing two 2.5 mm concave titanium spheres, two pink retention caps, a holder for placing the spheres on abutments, and a spatula for applying cement to the sphere (Figure 9).

We needed to fit concave spheres onto the worn abutment spheres in the mouth. To do this, we reduced the implant abutments with a bur before placing the spheres over the existing ball abutments. After achieving the required size, we attempted to fit the concave sphere onto the worn sphere within the oral cavity. For ease of handling, the sphere was placed in the designated sphere holder provided in the kit. The pink retention caps were placed over the cemented spheres, followed by placement and verification of the denture (Figure 9).

**Figure 10:**
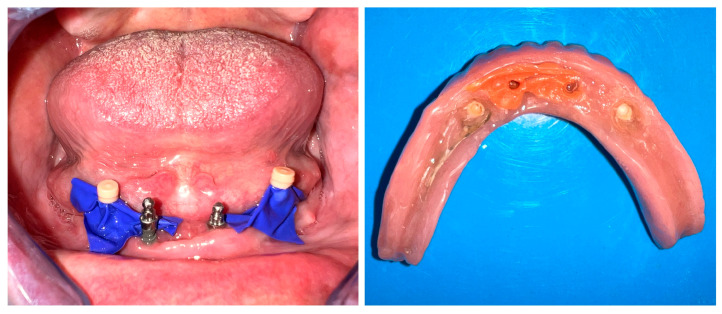
Blocking out all undercuts of abutments (**left**), pink retention caps secured in the overdenture (**right**).

The denture was adjusted to accommodate the new systems and current conditions, and the occlusion was evaluated. We applied primer to the metal spheres on the implant abutments to enhance metal-to-metal bonding, then positioned them firmly onto the existing balls. Dual-cure cement was used. After the cement set and the spheres were secured, we prepared the pink retention caps for placement in the overdenture base. All undercuts on the abutments were blocked out, and polymethyl methacrylate resin was used to fix the caps in the denture. The patient closed in maximal intercuspation during this step. The base of the overdenture was adjusted and placed in the patient’s mouth to accommodate the updated clinical situation and was checked and adapted for proper occlusion (Figure 10). The retention of the denture was improved, and patient satisfaction increased accordingly.

## 3. Discussion

Ball attachments are commonly used in mandibular implant overdentures due to their simplicity and affordability, yet wear of the abutments remains a frequent clinical challenge that can undermine prosthesis retention and patient comfort. Despite the prevalence of this issue, available literature offers limited guidance on how clinicians should assess and manage progressive wear.

Within this context, the present research evaluated clinical patterns of ball abutment deterioration and assessed the effectiveness of different management approaches. The findings indicate that timely replacement of worn components significantly improves overdenture stability, while consistent monitoring helps prevent advanced complications. These results highlight the need for standardized clinical protocols to optimize long-term outcomes in implant overdenture therapy.

Implant prosthetic solutions for complete mandibular edentulism are widely performed in clinical practice. According to the McGill and York Consensus (2002, 2009), overdentures supported by two implants are regarded as a standard treatment option for mandibular edentulism [8,9].

With advancements in biomaterials used for implantology and prosthetic components, this therapeutic procedure is commonly employed to enhance denture stability and provide functional benefits for patients. The procedure is minimally invasive, and mini-implants have high survival rates, making them a frequent option for improving the retention and functionality of overdentures [4,10].

In the case of a mandibular edentulous patient with a skeletal class II relationship, we implemented a prosthetic approach utilizing a mandibular overdenture supported by four interforaminally placed Mini^1^ SKY mini dental implants. This method enhances denture stability and mastication. The application of stage 1 mini-implants for overdenture stabilization offers a streamlined solution that generally yields favorable long-term outcomes. Various prosthetic abutments and retention systems are available, contributing to improved retention and increased masticatory efficiency.

Occlusal loading of dentures during mastication may lead to both biological and mechanical complications, including bone resorption around implants, peri-implantitis, mechanical failures of the prosthetic superstructures, and denture fractures, as well as wear or fracture of matrices and abutments. Bone loss tends to be more active during the initial five years, which may affect long-term follow-up regarding functional outcomes. Mechanical complications are also associated with the occurrence of biological complications. Abutment design affects denture stability and movement, potentially allowing hinge-like motion that leads to system wear over time. Additionally, a greater occluso-gingival height of distal abutments reduces prosthetic space, resulting in a thinner acrylic cap over the O-ring that is prone to deformation or fracture [10,11]. In these cases, alternative therapeutic solutions are recommended, such as overdenture systems utilizing two-piece implants with detachable abutments, or fixed prosthodontic approaches such as Fast & Fixed protocols [12,13].

The patient’s clinical progress has been favorable, particularly given the longevity of the mini-implants. However, over a 10-year period, multiple prosthetic rehabilitations have been required, with denture replacements necessitated by mechanical complications of prosthetic components and wear of the artificial teeth. In this case, various factors, including the relationship with antagonistic maxillary teeth (whether fixed prosthetic restorations or overdentures), persistence of mandibular anterior teeth, a tendency toward propulsion, and increased occlusal pressure—resulted in several prosthetic complications. These included denture fractures, wear of rubber rings, detachment of metal rings from the prosthesis, and wear on spherical abutments. Despite these challenges, the implants were maintained, offering superior functional outcomes compared to those achieved with a complete denture. This solution demonstrated satisfactory functional results initially; however, over time, wear of the ball abutment and challenges with denture retention have been observed, leading to increased difficulty in managing the long-term success of the prosthetic approach.

Retention of the denture was reduced due to wear of the spherical abutments and the inability to use O-ring systems. In this case, intraoral examination showed moderate bone resorption at the mandibular alveolar ridge around the mini-implants. The implants remained osseously stable despite ongoing medial resorption, with marginal bone loss observed around the implants as a biological response to masticatory and denture-related stresses.

The prosthetic option using silicone matrices or reconstructive spheric abutments serves to enhance retention and functionality. The main pitfalls of silicone matrices include their limited durability and tendency to deform under masticatory forces, resulting in decreased long-term retention and the need for more frequent replacement. In contrast, reconstructive cemented spheres depend on sufficient remaining abutment structure, precise surface preparation, and appropriate cement selection, with outcomes strongly influenced by operator technique. Additionally, both approaches may incur higher long-term costs due to maintenance and technique sensitivity. Goodacre et al. classified implant complications into six types: surgical, implant loss, peri-implant soft tissue, mechanical, esthetic, and phonetic issues. The authors reported that mechanical complications occur in about 30% of cases, such as loss of retention in implant overdentures, while implant fractures occur in 1% [14].

As highlighted in previous publications, there is a notable lack of research on the impact of mechanical complications on the prognosis of prostheses. Such studies could help identify vulnerable components and procedures contributing to implant failure. Nevertheless, mandibular overdentures retained by mini-implants are often considered an effective solution that results in high patient satisfaction [15].

In screw-retained prostheses, abutment damage has been reported as uncommon, with a prospective study by Zarb et al. noting a 1.1% occurrence. Another study, observing a 15-year follow-up period, found abutment damage reduced to 0%. In contrast, overdentures on mini implants with ball abutments have shown susceptibility to ball wear and potential loss of prosthesis retention [16].

Mechanical complications may arise after denture use but can be avoided through regular check-ups that assess denture stability, occlusal relations, and the need to replace matrices. Prosthetic maintenance and complications can vary based on the type of attachment system used. Patient satisfaction, however, does not necessarily depend on the type of attachment system [17].

Implant overdentures in the edentulous mandible demonstrate high survival rates across attachment types, with complications largely comparable, although prosthetic issues vary—greatest with magnets and lowest with LOCATORs or bars. Despite higher stress transmission from high-retention systems such as ball attachments, both ball and LOCATOR designs provide favorable tissue response, survival, and patient satisfaction, with peri-implant outcomes remaining similar between immediate and delayed loading protocols [13,18,19].

The denture base over the coping is subject to increased strain. Assuncao et al., in a finite element study, found higher stress concentration on the O-ring system capsule over the implants during loading. Regarding the supporting tissues, cortical bone exhibited the highest stress values [20].

The lateral movement of the mandible and denture can contribute to wear on the convexity of implant ball abutments, as observed in this case. Elsyad et al. found that ball attachments for implant-retained overdentures were associated with greater mandibular denture base deformation compared to locator attachments. Reinforcement of the denture base with ball attachments may be advisable to enhance the resistance of the overdenture base. As observed in our case and supported by previous clinical reports, fractures in overdentures frequently occur in the area surrounding abutments. Therefore, reinforcing this portion of the denture may be necessary to minimize the risk of such incidents. However, selecting a retention system that fits the prosthetic space is more important for preventing this incident [21,22,23,24].

Peri-implant microstrains vary according to mini-implant number, placement, and splinting pattern, with unilateral and elevated forces amplifying strain and four mini-implants configurations demonstrating the most favorable biomechanical behavior. Strategic increases in mini-implants number and distribution enhance overdenture stability and significantly improve masticatory performance across the first 12 months, particularly influencing anterior–posterior but not lateral resistance [25,26,27]. Studies indicate that overdentures retained by mini-implants improve mastication kinematics—such as reduced chewing time and fewer chewing cycles—thereby enhancing patients’ quality of life [28]. This aspect was observed in this case, particularly when the overdenture demonstrated effective retention and mastication [29].

Mandibular overdentures supported by mini-implants represent a prosthetic option with several benefits for edentulous mandibles. However, complications may arise due to patient anatomical and functional factors, characteristics of prosthetic components, and the duration of use. For optimal biomechanical function, implants should be placed parallel to each other. At the same time, the type of attachment may influence potential denture movement and the distribution of stress on both bone and implants [30].

Several authors have concluded that ball abutments and locator systems are among the most effective overdenture options, given their favorable tissue response, high survival rates, and enhanced patient satisfaction [13]. Over a 10-year period, the patient with a skeletal class II relationship experienced various biomechanical and functional issues, including the wear of rubber rings, loss of some matrices, denture fractures, and notable clinical wear of ball abutments. These factors contributed to reduced retention and stability of denture, as well as decreased functionality and comfort. The case report describes mechanical complications that, while not affecting implant survival, present challenges in finding alternative retention systems for dentures on worn implant abutments.

3D-printed implant-supported overdenture materials exhibit the highest flexural strength, whereas conventional and high-impact heat-cured acrylics show comparable mechanical properties. Reinforcing the overdenture with a metal framework—particularly digitally fabricated designs that integrate the attachment housing—enhances rigidity, strengthens the denture base, and reduces fracture risk around implant abutments [31,32,33,34].

These approaches offer prosthetic solutions that utilize advanced materials and devices to improve the balance and function of mandibular overdentures supported by mini-implants, particularly in edentulous patients with a class II skeletal relationship [35].

The periodic replacement of the silicone matrix presents certain disadvantages, particularly in terms of longevity. An extended solution involves the reconstruction of the abutment on the dental implant by utilizing a prefabricated component, such as the reconstructive sphere, to address ball wear. Employing reconstructive spheres cemented onto worn abutments serves as a longer-term prosthetic alternative, offering improved denture retention, enhanced functionality, and increased comfort. When considering a successful alternative, it is essential to evaluate both the biomechanical performance under masticatory loading and factors such as optimal retention and patient satisfaction.

The present case report has several limitations that should be acknowledged. The sample size and observation period may not fully capture long-term mechanical behavior or late complications associated with reconstructive spheres and silicone matrices. The focus on a single attachment system and specific clinical protocol may also restrict the extrapolation of results to other implant systems, attachment designs, or patient populations with different levels of residual ridge resorption, functional aspects or parafunctional habits.

Future research should include an extended follow-up to validate the durability and reliability of reconstructive spheres as a long-term solution for worn abutments. Comparative studies of different attachment systems, matrix materials, and reconstructive techniques are needed to refine selection criteria and optimize biomechanical performance under functional loading. Integrating digital analysis of wear and finite element modeling could further clarify the relationship between mechanical behavior, retention, and perceived comfort. Such investigations would support the development of evidence-based clinical protocols and broaden the application of these minimally invasive, patient-centered strategies in geriatric implant prosthodontics.

## 4. Conclusions

In conclusion, this case report of a patient with a class II skeletal relationship and a mandibular overdenture supported by mini-implants with an O-ring retention system highlights specific mechanical and functional complications. These complications are attributed to anatomical and functional factors, which arise from skeletal relationships and, more specifically, from the prosthetic construction of overdentures supported by mini-implants with ball abutments and an O-ring retention system.

The longevity of the implants was not compromised, even though the denture functioned effectively for more than 14 years. However, progressive wear of the ball abutments over time presented challenges for subsequent rehabilitations, necessitating the consideration of alternative methods to enhance denture retention on the worn implant abutments and thereby improving functionality. By using silicone matrices and reconstructive cemented spheres on worn abutments, we replaced the O-ring systems and adapted the retention system, preserving a prosthetic solution superior to a conventional complete denture in this case. This study aimed to characterize the clinical consequences of abutment wear, evaluate alternative management strategies, and provide evidence-based guidance for optimizing retention in aging overdenture systems.

Clinically, these findings underscore the importance of early detection of attachment wear, systematic follow-up appointments, and individualized selection of alternative retention solutions when conventional components are no longer viable. Future clinical practice may benefit from standardized protocols for evaluating attachment wear, greater emphasis on minimally invasive rehabilitative options, and integration of patient-centered outcomes—particularly in elderly populations who rely heavily on overdenture stability for oral function and quality of life.

## Data Availability

The raw data supporting the conclusions of this article will be made available by the authors on request.
